# Dr. Robert Newland Blanchard

**Published:** 1918-04

**Authors:** 


**Dr. Robert Newland Blanchard,** University of Buffalo, 1881, died in Jamestown, Jan. 18, age 62.

